# Stoichiometry of HLA Class II-Invariant Chain Oligomers

**DOI:** 10.1371/journal.pone.0017257

**Published:** 2011-02-22

**Authors:** Norbert Koch, Martin Zacharias, Angelika König, Sebastian Temme, Jürgen Neumann, Sebastian Springer

**Affiliations:** 1 Division of Immunobiology, Institute of Genetics, University of Bonn, Bonn, Germany; 2 Physik Department, Technische Universität München, Munich, Germany; 3 Molecular Life Science Center, Jacobs University Bremen, Bremen, Germany; Universidade de Sao Paulo, Brazil

## Abstract

**Background:**

The HLA gene complex encodes three class II isotypes, DR, DQ, and DP. HLA class II molecules are peptide receptors that present antigens for recognition by T lymphocytes. In antigen presenting cells, the assembly of matched α and β subunits to heterodimers is chaperoned by invariant chain (Ii). Ii forms a homotrimer with three binding sites for class II heterodimers. The current model of class II and Ii structure states that three αβ heterodimers bind to an Ii trimer.

**Methology/Principal Findings:**

We have now analyzed the composition and size of the complexes of class II and Ii using epitope tagged class II subunits and density gradient experiments. We show here that class II-Ii oligomers consist of one class II heterodimer associated with one Ii trimer, such that the DR, DQ and DP isotypes are contained within separate complexes with Ii.

**Conclusion/Significance:**

We propose a structural model of the class II-Ii oligomer and speculate that the pentameric class II-Ii complex is bent towards the cell membrane, inhibiting the binding of additional class II heterodimers to Ii.

## Introduction

Major histocompatibility complex (MHC) class II (MHCII) peptide receptors are sensors on antigen presenting cells (APC) that alert the adaptive immune system to the presence of foreign antigens by presenting them to CD4^+^ T cells. To achieve presentation of a large variety of antigenic peptides, the human MHC (human lymphocyte antigen, HLA) contains three isotypic loci, DP, DQ, and DR, each of which encode the α and β subunits that form heterodimeric MHCII receptors. An extremely high polymorphism of the MHCII genes creates a large diversity of the receptors.

Following biosynthesis, the MHCII subunits associate with the chaperone invariant chain (Ii). Ii forms a homotrimer (Ii_3_) [1]–[3], and MHCII glycoproteins bind to a sequence of Ii that occupies the peptide binding groove of the αβ heterodimer [4]–[6]. Additional interaction sites of MHCII and Ii have been demonstrated [7]–[11]. The homotrimeric structure of Ii would suggest that three MHCII heterodimers assemble with an Ii trimer to form a nonameric complex, Ii_3_(αβ)_3_. Indeed, this assumption was supported by chemical crosslinking experiments with MHCII complexes that showed a high molecular weight band with an apparent size of 260 kDa [12]–[13]. The oligomeric assembly of MHCII and Ii is a prerequisite for intracellular transport to endosomes, followed by degradation of Ii and subsequent acquisition of exogenous peptides [14].

One possible consequence of the nonamer model is that one complex could contain different DP, DQ, or DR isotypes bound to an Ii homotrimer. Because of technical difficulties involved in distinguishing the individual isotype subunits, this obvious prediction has not been conclusively tested to date. Remarkably, initial experiments in mice have shown exclusive co-isolation of Ii with the murine MHCII isotypes H-2A or H-2E, but no co-isolation of these two isotypes with each other [15]. Based on this observation, we have now examined whether indeed DP, DQ, and DR are found in the same oligomer with Ii.

In contrast to the MHCII heterodimers, Ii has a short cellular half-life [16]–[17]. Apparently as a compensation, Ii is synthesized at a significantly higher rate than MHCII, which leads to a large excess of the Ii polypeptide in the endoplasmic reticulum (ER) [18]. Since a stochastic assembly of MHCII subunits with Ii would therefore yield mostly pentameric complexes (with one MHCII heterodimer per Ii_3_), the formation of Ii_3_(αβ)_3_ nonamers would require cooperative binding of MHCII αβ heterodimers to Ii_3_. However, *in vitro* studies with soluble MHCII and Ii have suggested that complex formation is not sufficiently cooperative [19].

In recent years, novel methods have been employed to assess the stoichiometry of the murine γδ T cell receptor and of the B cell antigen receptor [20]–[21]. These reports have yielded unpredicted results regarding the ratio of the antigen receptors to their associated signalling molecules and have thus led to the revision of previous models.

In this work, we have employed epitope-tagged MHCII molecules to identify individual subunits of MHCII-Ii oligomers and to examine their stoichiometry. In transfected APCs, we have also studied the association of the tagged MHCII subunits with endogenous MHCII chains. We find that DP, DQ, and DR molecules are not co-isolated from cell extracts, which suggests that these MHCII isotypes are not simultaneously contained in one complex with Ii. In a parallel approach, immunoprecipitation of DQ from separated MHCII-Ii complexes did not result in isolation of additional DR heterodimers as the nonamer model would suggest. To directly determine the size of DR-Ii complexes, we have estimated the size of the oligomer by ultracentrifugation on a sucrose gradient to be approximately 130 to 160 kDa. We thus present a pentamer model where only one single DR, DQ, or DP heterodimer is contained in a MHCII-Ii complex and propose a conformational change induced upon binding of one MHCII heterodimer to the Ii_3_ that could explain why class II molecules do not abundantly occupy all three CLIP sequences contained in the Ii_3_ complex.

## Results

### Size separation of DR and of Ii complexes

**Figure 1:**
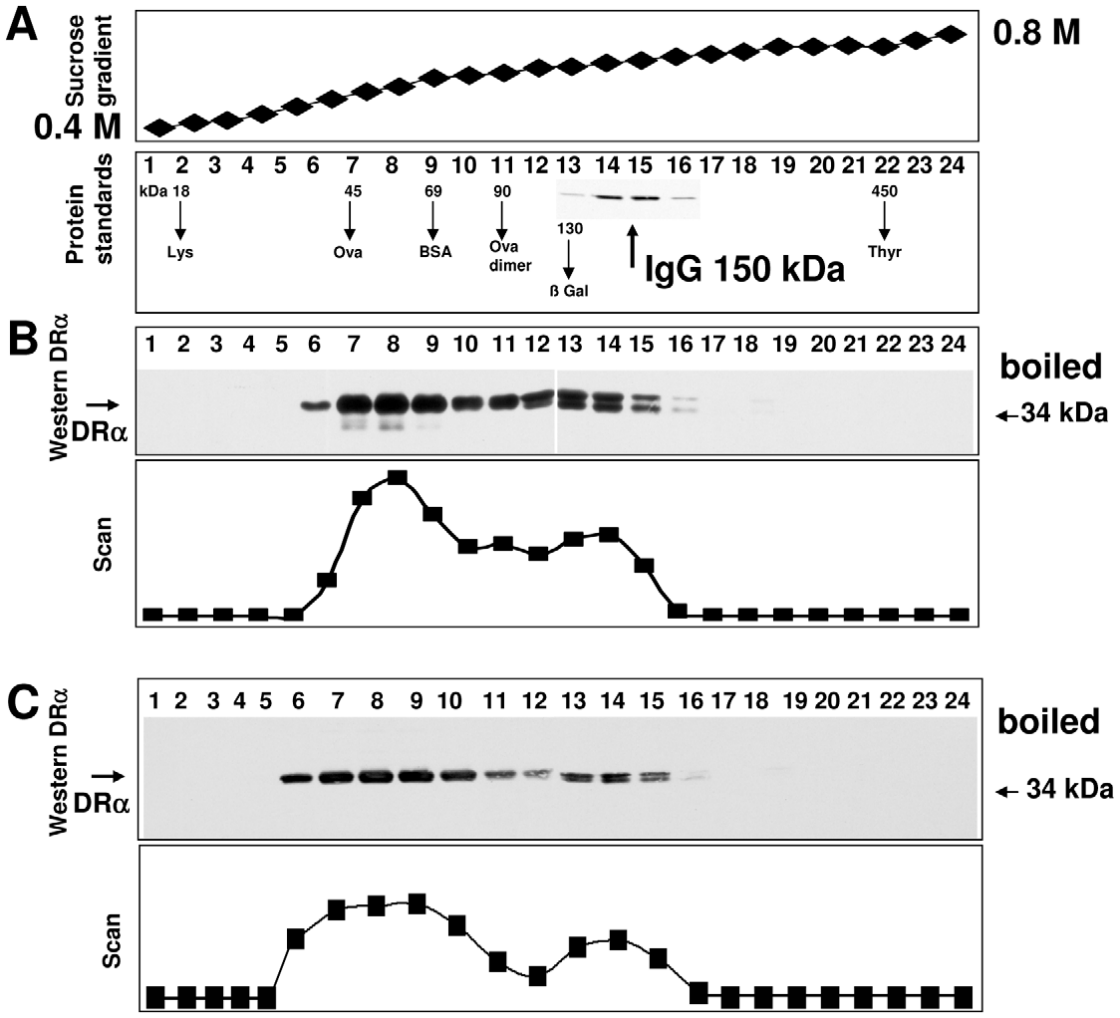
Separation of cell extracts on a sucrose gradient by ultra-centrifugation. Cell lysates were ultra-centrifugated on a 0.4 to 0.8 M sucrose gradient at 100 000 g for 50 h. A) The sucrose concentrations of the fractions were determined after ultracentrifugation by a refractometer (upper panel). In a parallel tube, molecular weight standards were separated (lower panel). The SDS-PAGE separated protein standards were stained with Coomassie blue. The peak positions of the separated molecular weight markers are indicated by arrows. IgG bands (insert) are shown in fractions 14 and 15. B) Twenty four samples, obtained from the fractions of the sucrose gradient of Raji cell lysates were boiled in SDS sample buffer and analysed by SDS-PAGE and subsequent western blotting with monoclonal antibody TAL-1B5 against DRα (upper panel). C) Cell lysates from monocyte-derived human dendritic cells separated by the sucrose gradient were western blotted for DRα. The positions of DRα and of a protein standard (34 kDa) are indicated by arrows. The lower panels of B and C show densitometric scans of the western blots.

In order to assess the molecular weight of cellular complexes of endogenous Ii and MHCII molecules, we prepared lysates from Raji cells, separated the solubilized proteins on a sucrose density gradient, and detected DRα by western blotting. The DRα band appeared in fractions 6 to 16 (Fig. 1B). A densitometric scan of the blot shows two peaks (Fig. 1B, lower panel) that correspond to molecular weights of 40–70 (fractions 7 to 9) and of 130–160 kDa (fractions 13 to 15), respectively, as determined by protein standards (Fig. 1A). The double band seen in fractions 12 to 16 of Fig. 1B is due to differential carbohydrate processing of the DRα glycoprotein, which was assessed by EndoH treatment of the DRα glycoprotein (data not shown). The lower band represents the endoplasmic reticulum (ER) form of the MHCII subunit, and the upper band contains DRα with complex type carbohydrates.

In order to test whether separation of MHCII complexes is also achieved in primary cells, we used lysates of monocyte-derived dendritic cells (DC) for separation in a sucrose gradient. Fig. 1C shows a western blot of 24 fractions of sucrose gradient separated DC lysates. The scan of the DRα western blot reveals two peaks, similar to the result obtained with Raji cell extracts in Fig. 1B.

**Figure 2 pone-0017257-g002:**
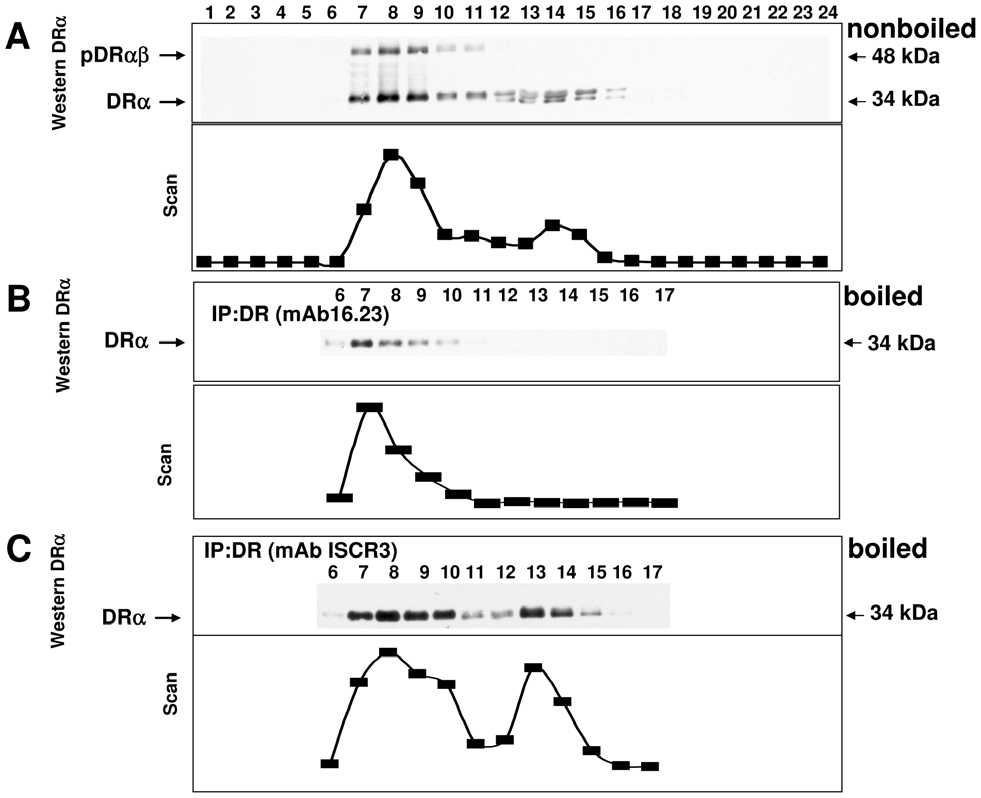
SDS-PAGE separation of Raji cell extracts from sucrose gradient fractions. The lower panels depict densitometric scans of the western blots. A) Samples treated with SDS sample buffer at room temperature were SDS-PAGE separated and immunoblotted for DRα (monoclonal antibody TAL-1B5). The upper panel shows SDS resistant pDRαβ bands (ca. 55 kDa) in fractions 6 to 11. The Scan represents both the pDRαβ and the DRα bands. B) Immunoprecipitation (IP) of DRαβ with monoclonal antibody 16.23, and western blotting for DRα (upper panel). The monoclonal antibody 16.23 selectively immunoisolates DR from fraction 6 to 10 and shows no reactivity to DR in fractions 11 to 17. C) Immunoprecipitation from sucrose gradient fractions 6 to 17 with DR monoclonal antibody ISCR3. SDS-PAGE separation and western blotting for DRα (monoclonal antibody TAL-1B5) results in separation of two DR populations, which is visualized as two separate curves in the density scan.

To assess which one of the two peaks in Fig. 1B contained peptide-bound DR molecules, fractions were treated with sodium dodecyl sulphate (SDS) at room temperature prior to SDS-PAGE, since DR heterodimers with a bound peptide resist SDS-induced dissociation under these conditions [22]. An SDS-resistant band at ca. 55 kDa, corresponding to peptide-loaded DRαβ complexes (pDRαβ), was indeed observed in fractions 7 to 9 (Fig. 2A). No pDRαβ band was detected beyond fraction 11, indicating that this range of the gradient contains no peptide-loaded DR heterodimers. This suggests that fractions 13 to 15 contain the complexes of peptide-free DR with Ii. To further examine the composition of the separated DR complexes, we next subjected the gradient fractions to immunoprecipitation with anti-DR monoclonal antibodies. The anti-DR monoclonal antibody 16.23, which reacts with DRαβ heterodimers but not with DR-Ii oligomers [23], only immunoprecipitates DR from fractions 7 to 9 (Fig. 2B). In contrast, the conformation-dependent monoclonal antibody ISCR3 immunoprecipitates DR from both peaks (Fig. 2C), demonstrating the presence of DRαβ dimers.

**Figure 3:**
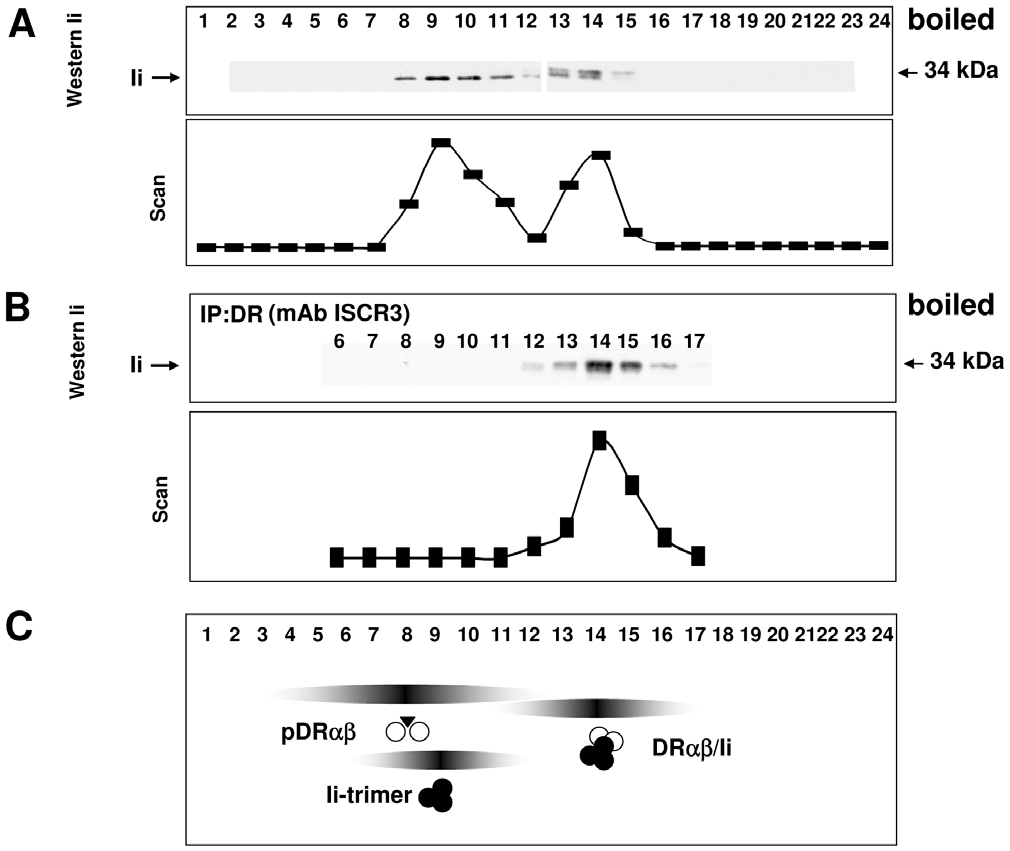
Detection of Ii in sucrose gradient fractions and delineation of DR and Ii complexes. A) SDS-PAGE separated Raji cell extracts from fractions 1 to 24 and western blotting for Ii (monoclonal antibody Bu43) (upper panel) results in two peaks (scan lower panel). B) Immunoprecipitation of DR, and SDS-PAGE separation was conducted as in Fig. 2C, followed by immunoblotting for Ii (monoclonal antibody Bu43, upper panel) and evaluation as a scan (lower panel). The DR-associated Ii appears in lanes 12 to 16. C) DR and Ii oligomers displayed in a sceme. Peptide-loaded DR (pDRαβ) largely co-migrates in the sucrose gradient with the Ii trimer. Complexes containing DR-Ii are separated in fractions 12 to 16.

We next probed SDS-PAGE separated fractions for Ii and again found two peaks, in fractions 8 to 11 and 13 to15 (Fig. 3A). In contrast, blotting of the DR immunoprecipitates for Ii (Fig. 3B) resulted in a single peak at fractions 13 to15, which therefore contains the DR-Ii oligomer. No signal for Ii was obtained in fractions 6 to 11, suggesting that in the low molecular weight range of the gradient, Ii is present but not associated with the DRαβ heterodimer. Immunoprecipitation of sucrose gradient separated DC lysates with a monoclonal antibody to DR and subsequent blotting for Ii showed again that Ii was only detected in fractions 13 to 16 (data not shown), indicating that these fractions contain DR-Ii complexes.

The results of the sucrose gradient experiments are summarized in Fig. 3C. The pDRαβ complex displays the lowest mobility of the examined complexes (fractions 7 to 9). The low molecular weight peak of the Ii western blot (Fig. 3A), is found in fractions 8 to 11, slightly heavier than the pDRαβ complex (Fig. 2A) and it may represent Ii_3_. The peaks of pDRαβ and Ii_3_ overlap to a great extent, since molecules of this size are not completely separated on this sucrose gradient. Crucially, however, even though DR and Ii are both present in this region of the gradient, no interaction between them can be detected. The high molecular weight peak of DR in Fig. 2C and the peak of the DR-Ii complex in Fig. 3B both most likely represent the DRαβIi oligomer. This complex comigrates with immunoglobulin G (150 kDa), which suggests that is significantly smaller than the estimated molecular weight of the postulated nonameric complex (DRαβ)_3_(Ii)_3_, 260 kDa [12]. Remarkably, the amount of oligomeric DR-Ii is lower than the amount of DRαβ heterodimers. From the density scans in Fig. 1B and Fig. 2C, we estimate that under the steady state conditions of this experiment, roughly one third of the DR molecules in the cell are contained in DR-Ii complexes.

### Assessment of DR and Ii oligomers by chemical crosslinking

**Figure 4:**
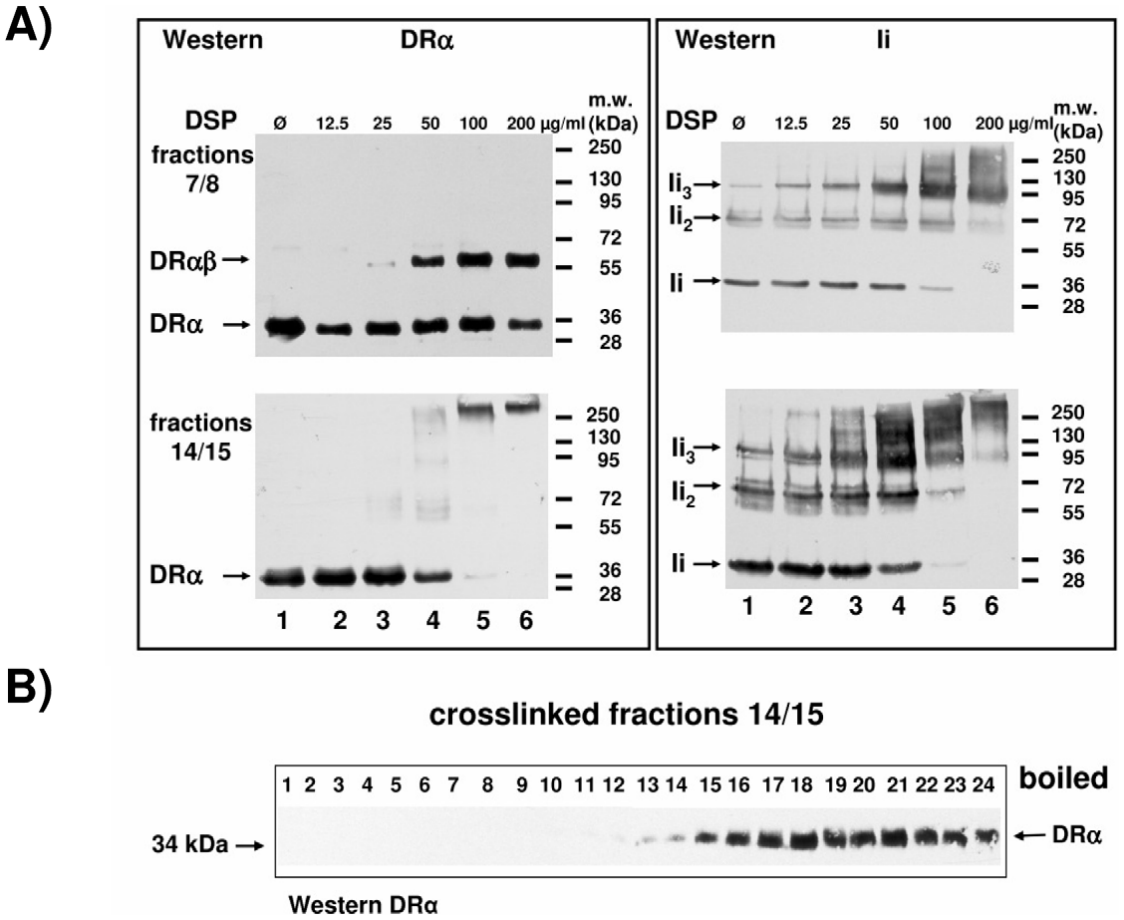
Chemical crosslinking of DR and of Ii oligomers. DR and Ii oligomers separated by sucrose gradient fractions of Raji cell lysates were crosslinked with DSP. A) Sucrose gradient fractions 7/8 and fractions 14/15 were analysed in the upper or lower panels. Concentration of DSP is shown on top. Untreated or crosslinked samples were separated by SDS-PAGE and western blotted for DRα (monoclonal antibody TAL-1B5, left panels) or for Ii (monoclonal antibody Bu43, right panels). On the right, positions of molecular weight markers are depicted. The dimeric form of Ii (70 kDa), which is seen both in untreated and in crosslinked samples, is produced by oxidation of a free cysteine residue of Ii to an inter-molecular disulfide bond [24]. B) Fractions 14/15 were crosslinked with 200 µg DSP/ml. Subsequently, crosslinked proteins were subjected to ultra-centrifugation on a sucrose gradient, separated by reducing SDS-PAGE followed by immunoblotting for DRα.

To demonstrate association between DR and Ii in an independent experimental system, Raji cell lysates were separated on a sucrose gradient, and samples from the low and from the high molecular weight peaks of DR in Fig. 1B were treated with the chemical crosslinker, 3,3′- dithiobis [succinimidyl propionate] (DSP) and separated by SDS-PAGE followed by immunoblotting for DRα or for Ii (Fig. 4A). At low concentrations of DSP, DRα from fractions 14/15, which contain the DR-Ii complex, remains monomeric, but at 100 and 200 µg/ml DSP, it shifts to a high molecular weight band that appears on top of the gel (Fig. 4A bottom left panel).

The crosslinking of DR from fractions 7/8 (which correspond to peptide-bound DR molecules) with increasing concentration of DSP converts the DRα monomer into a band that has the expected size of the DRαβ dimer (55 kDa; Fig. 4A, top left panel). No other bands were detected suggesting that DR crosslinking in these fractions is specific and does not result in the crosslinking of DR to other cellular proteins. In striking contrast, blotting of the same crosslinked fractions for Ii yielded a smear of high molecular weight material at the top of the gel at 100 and 200 µg/ml of DSP, while monomeric, dimeric, and trimeric Ii appear as distinct bands (top and bottom, right panels). With increasing DSP concentration, Ii_3_ (95 kDa) becomes abundant, and in fractions 14/15 (which contain the DR-Ii complex), high molecular weight material accumulates above the Ii trimer and, at 200 µg/ml DSP, appears as a compact band at the very top of the gel. Importantly, we noted that even in the absence of association with DR, in fractions 7/8 (upper right panel), high molecular weight crosslinks of Ii were formed. This demonstrates that Ii associates with cellular components other than MHCII, as described previously [25]–[30]. Since such additional Ii-interacting proteins may be contained in all Ii-complexes that are obtained by chemical crosslinking, it must be assumed that the crosslinked high molecular weight material that is detected by immunoblotting for DR and for Ii at the top of the gel (Fig. 4A, lower panels) does not solely consist of DR and Ii but contains other cellular proteins.

To determine the size of the high molecular weight band that was obtained upon crosslinking of the DR-Ii complexes, we separated the crosslinked samples by ultracentrifugation on a sucrose gradient. We found that DRα bands did not correspond to a species of uniform size but were almost evenly distributed all across the high molecular weight area of the gradient (Fig. 4B). This demonstrates that chemical crosslinking produces DR complexes with larger size than obtained by separation of untreated cell lysates in a sucrose gradient (compare Fig. 4B to Fig. 1B). Presumably, unstable and transient interactions of Ii with cellular components are stabilized by the crosslinking reagent. This might have led previous workers to propose a larger apparent size of the DR-Ii complex.

### HLA- DR, DP, and DQ heterodimers segregate into individual complexes with Ii

**Figure 5:**
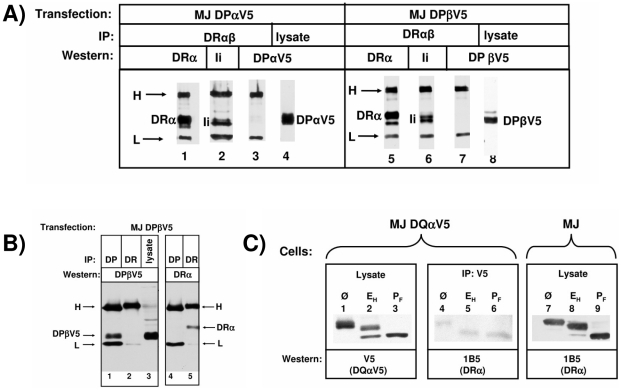
Inspection for co-isolation of DP and DQ isotypes with DR. A) MelJuSo cells were transiently transfected with cDNAs encoding V5-tagged DPα or DPβ encoding cDNAs. Cells lysed in 1% NP40 were immunoprecipitated for DRαβ (monoclonal antibody ISCR3) (lanes 1,2,3,5,6 and 7). Lanes 4 and 8 contain cell lysates. The SDS-separated polypeptides were immunoblotted for DRα (lanes 1 and 5), for Ii (lanes 2 and 6), and for DPαV5 (lanes 3 and 4), or for DPβV5 chains (lanes 7 and 8). The position of DRα, Ii and Ab-epitope tagged DPαV5 or DPβV5 bands is indicated. H and L chain bands derived from the Ab used for immunoprecipitation are labeled on the left. B) Immunoprecipitates of the V5-tagged DPβ (lanes 1 and 4) and of DRαβ (lanes 2 and 5) chains were separated by SDS-PAGE and either blotted for DPβV5 (lanes 1 to 3) or for DRα (lanes 4 and 5). To monitor expression of DPβV5, lane 3 contains cell lysate. The position of DPβV5, of DRα and of H and L Ig chains are indicated. C) MelJuSo cells were transfected with V5-tagged DQα cDNA. Cell lysate was immunoprecipitated for the V5-tagged DQα chains. Immunoprecipitates (lanes 4 to 6) and cell lysates (lanes 1 to 3) were digested with EndoH (E_H_), or with PNGase F (P_F_) or left untreated (Ø), separated by SDS-PAGE and western blotted as indicated. In lanes 7 to 9 MelJuSo cell lysates untreated and glycosidase treated were western blotted for DRα.

If Ii_3_ associates with more than one MHCII dimer, one would assume that such complexes contain a mixture of MHCII isotypes and that co-isolation of the Ii-bound forms of MHCII with each other should be possible. In contrast, if Ii_3_ and MHCIIαβ form pentameric complexes only, such co-isolation should not be possible. We therefore transiently transfected the MHCII- and Ii-expressing human melanoma cell line MelJuSo with cDNA encoding epitope-tagged DPα or DPβ chains, lysed the cells and immunoprecipitated the endogenous DRαβ heterodimer. The immunoprecipitates and cell lysates were separated by SDS-PAGE and immunoblotted for the presence of transfected epitope-tagged DPα or DPβ, or as a control for endogenous DRα and Ii. No co-isolation of DPα or DPβ with DRα was detected, suggesting that different MHCII isotypes are not associated via Ii (Fig. 5A, lanes 3 and 7).

In the next experiments, we again examined MHCII-Ii complexes for the presence of two different MHCII isotypes. To achieve a uniform expression of the transfected cDNA and to make sure that the transfected MHCII subunits are integrated into endogenous MHCII-Ii complexes and introduced into the biosynthetic route of MHCII molecules, we used stably transfected cell lines. We first demonstrated by carbohydrate maturation, endosomal localization, and formation of SDS-resistant dimers that the transgenic MHCII subunits are assembled and transported together with endogenous MHCII (see Text S1, supporting information, Fig. S1A, B, C and D). Next, we tested the sensitivity of the co-isolation experiment. We immunoprecipitated the transfected V5-tagged MHCII chain and immunoblotted for co-isolated Ii. The result indicates that a substantial amount of Ii was co-isolated with the V5-tagged MHCII chain (Fig. S1E), and it suggests that any additional MHCII isotype heterodimers (which should be present in the same complex according to the nonamer model) should be readily detectable. Since this was not the case in the above experiment (Fig. 5A), we conclude that in our experimental system, nonameric Ii_3_(αβ)_3_ complexes with mixed isotypes most likely do not exist.

To make sure that a potential nonameric complex of MHCII and Ii had not been partially disrupted by the use of the detergent Triton X-100, we decided to test the stability of MHCII-Ii complexes in other detergents, especially the mild detergent digitonin. Ii was immunoprecipitated from a digitonin cell lysate and bound to Protein G Sepharose. This Ii-immunoprecipitate (which according to the nonamer model, should contain three MHCII heterodimers and Ii_3_) was then incubated with Triton X-100 to test whether Triton X-100 would release any MHCII proteins. The immune complexes on the beads and the Triton X-100 supernatant were then separated on SDS PAGE and immunoblotted for Ii and for DRα. Fig. S1F shows that Ii and DRα were co-isolated in the immunoprecipitate, but that no DRα was detected in the Triton X-100 supernatant. This result indicates that Ii-DR complexes were stable in Triton X-100 and that in digitonin and Triton X-100 lysates, identical amounts of DR are associated with Ii. Thus, Triton X-100 induced dissociation is not an explanation for the lack of detection of nonameric Ii_3_(αβ)_3_ complexes.

According to the nonamer model, it should be possible to co-immunoprecipitate different MHCII isotypes that are bound to Ii in the nonameric complex directly with each other. To test this assertion, we next examined whether endogenous DR could be precipitated from the lysate of MelJuSo in a complex with stably expressed DPβ tagged with the V5 epitope (DPβV5). DP or DR molecules were immunoprecipitated and immunoblotted for DPβV5 or for DRα, respectively. We found that immunoprecipitation of DP and a subsequent western blot for V5 detects DPβV5 (Fig. 5B, lane 1), but not DRα (lane 4; correspondingly, the DR immunoprecipitate shows a band for DRα (lane 5), but not for DPβV5 (lane 2). These results suggest that transfected DPβ and endogenous DR chains are not found in the same protein complex in cells.

Next, we studied the co-isolation of transgenic DQα with endogenous DRα, hypothesizing that if more than one isotypic heterodimer is contained in a nonameric complex with Ii, it should be possible to co-isolate two isotypic α chains. We transfected MelJuSo cells with a cDNA encoding V5-tagged DQα and, to examine the functional assembly of this transgenic DQα with the endogenous β chain, we inspected the maturation of the DQα chain glycans. DQα chain contains two N-linked carbohydrates (upon arrival of the MHCII oligomer in the medial Golgi apparatus, one of the two glycans of the α chain acquires endoglycosidase H (EndoH) resistance, whereas the other remains EndoH sensitive). We found that most of DQα acquired partial EndoH resistance (Fig. 5C), which demonstrates that the transiently expressed DQα is transported out of the ER.

To assess whether endogenous DRα co-isolates with the transfected DQαV5, DQαV5 was then immunoprecipitated with a monoclonal antibody against the V5 epitope. Western blotting of the immunoprecipitate showed a faint band of endogenous DRα (Fig. 5C, lane 4), which was completely sensitive to EndoH treatment (lane 5), indicating that these aggregates of DQ and DR were transient and did not leave the ER. It is important to emphasize that the amounts of DRα co-isolated with DQαV5 are traces of bands at the limit of detection.

For comparison to lanes 1 to 3, glycosidase-treated lysates of MelJuSo cells were blotted for DRα (lanes 7 to 9). In conclusion, the results in Figure 5 indicate that the post-ER forms of DR, DQ and DP glycoproteins are not co-isolated from MelJuSo cell lysates, and that therefore, the different isotypes most likely form entirely separate complexes with Ii.

### DRαβ heterodimers form pure isotype complexes with invariant chain

The existence of such isotype-specific complexes of MHCII and Ii could be explained in two different ways. Either, an Ii homotrimer would form a complex with two or three MHCII heterodimers from one particular isotype only. Alternatively, one Ii homotrimer would only ever bind one MHCII heterodimer in total, of whatever isotype, to form a pentameric complex.

**Figure 6:**
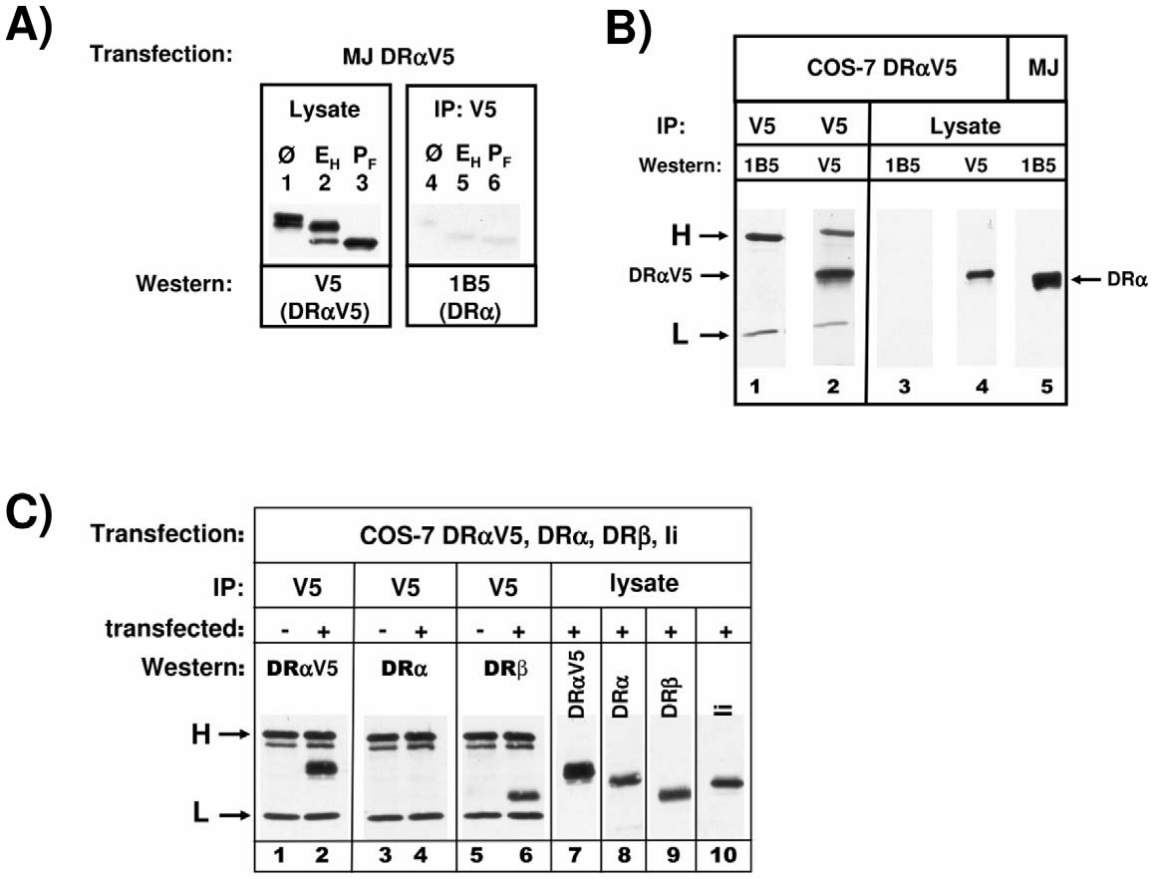
Isolation of Ab-epitope tagged DRα chains from transfected MelJuSo cells. A) MelJuSo cells transfected with DRαV5 cDNA were lysed. Cell lysates and V5-immunoprecipitates from cell lysates were digested with EndoH (E_H_) (lanes 2 and 5), with PNGse F (P_F_) (lanes 3 and 6), or left untreated (Ø) (lanes 1 and 4). SDS-PAGE separated cell lysates were immunoblotted for DRαV5 (monoclonal antibody V5) and V5-immunoprecipitated for DRα (western blotted with DRα monoclonal antibody TAL-1B5). B) Reactivity of monoclonal antibody TAL-1B5 to V5-tagged DRα is abolished. COS-7 cells were transfected with DRαV5 encoding cDNA. Cell lysate was immunoprecipitated with V5 or with TAL-1B5 (DRα) monoclonal antibodies. Immunoprecipitates were separated in lanes 1 to 4, and lane 5 contains lysate of MelJuSo cells. Lanes were western blotted as indicated. C) COS-7 cells were transfected with DRαV5, DRα, DRβ and Ii encoding cDNAs. Cell lysates from transfected (+) and from non-transfected (−) COS cells were immunoprecipitated for V5 (lanes 1 to 6). Immunoprecipitates and cell lysates (lanes 7 to 10) were SDS-PAGE separated and western blotted with mAbs V5 for DRαV5, TAL-1B5 for DRα, LGII-612.14 for DRβ, and Bu43 for Ii.

To distinguish between these two possibilities, we next investigated whether the transgenic DRαV5 chain is found in the same complex with Ii and with endogenous DRα chain. We hypothesized that if this complex contains several DR heterodimers, it should be possible to detect two DRα chains in a complex with Ii. We therefore transfected MelJuSo cells with a V5-tagged DRα chain. Inspection of carbohydrate maturation showed that the majority of transfected DRα chains was partially EndoH resistant, indicating that they were assembled with endogenous β chains and transported beyond the *cis*-Golgi (Fig. 6A). To distinguish between endogenous DRα and transfected DRαV5, we used the DRα-specific monoclonal antibody TAL-1B5, whose epitope is at the extreme C-terminus of DRα [31]; the extension of the C-terminus of DRα by the V5 epitope abrogates TAL-1B5 binding (Fig. 6B, lanes 1 and 3). When DRαV5 was immunoprecipitated with the anti-V5 antibody and immunoprecipitates were blotted for co-isolated endogenous DRα with TAL-1B5, only very faint traces of endogenous DRα were detected (Fig. 6A, right panel). These very faint bands were entirely sensitive to EndoH treatment, suggesting weak and transient interactions between endogenous DRα and transfected DRαV5 in the ER only, but not at later stations of the intracellular pathway.

To demonstrate separation between endogenous DRαand transfected DRαV5 in DR-Ii complexes by an independent experimental approach, we transiently expressed DRαV5, DRα, DRβ, and Ii simultaneously in COS-7 cells (Fig. 6C). DRαV5 was immunoprecipitated from transfected and from untransfected COS cells (lanes 1 to 6) and immunoblotted for DRαV5, DRα and DRβ. (As a control, the expression of the transfected cDNAs is shown in lanes 7 to 10). As before, no DRα was co-immunoprecipitated with DRαV5 (lane 4), while the DRαV5 chain (lane 2) and the DRβ chain (lane 6) were readily detected in the V5 immunoprecipitates. Taken together, these experiments demonstrate that DRα and DRαV5 are contained in separate complexes, and that Ii-DR complexes inside cells contain only one DRαβ heterodimer.

An analogous experiment with DRβ chain showed no co-isolation of endogenous DRβ with DRβV5, confirming the observation with DRα (see Text S1, supporting information, Fig. S2A). In addition, this experiment was repeated with the detergent digitonin (Fig. S2B). Again, endogenous DRβ was not co-isolated with DRβ-V5.

**Figure 7:**
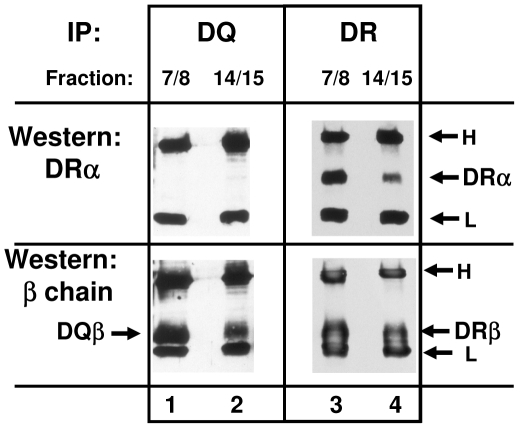
Isolation of DQ-Ii complexes from sucrose gradient fractions. Sucrose gradient fractions of Raji cell lysates were subjected to immunoprecipitation with mAb 1a3 against DQ and with DR mAb ISCR3. Fractions 7 and 8 or 14 and 15 were pooled and immunoprecipitated. DQ (lanes 1 and 2) or DR (lanes 3 and 4) were isolated, and western blotted for DRα (upper panel, mAb TAL-1B5) or for DQβ (lower panel, lanes 1 and 2) and for DRβ (lanes 3 and 4) with mAb LGII-612.14. The position of DRα, of DQβ or of DRβ and bands corresponding to immunoglobulin heavy (H) and light (L) chains are labeled by arrows.

To exclude the possibility that the observed pentameric complexes were an artefact of transfection, and to verify that under non-transfected conditions DR and DQ segregate into individual complexes with Ii, we employed the mAb 1a3, which reacts with DQ but does not crossreact with DR. We lysed untransfected Raji cells and separated the lysates on sucrose gadients as before. In these gradients, the pooled fractions 7/8 contain peptide loaded MHCII heterodimers (e.g. DQαβ and DRαβ heterodimers), and fractions 14/15 harbour MHCII-Ii complexes (see Fig. 1 to [Fig pone-0017257-g002] 3). Both pools of fractions were immunoprecipitated for DQ (Fig. 7, lanes 1 and 2) and for DR (lanes 3 and 4) and subsequently immunoblotted for DRα (upper panels) or for DQβ or DRβ chains (lower panels). Immunoprecipitation of DQ from fractions 7/8 (lane 1) shows no reaction with the DRα mAb (upper panel), confirming that mAb 1a3 does not crossreact to DR. Staining of DQβ (lower panel, lane 1) indicates that 1a3 immunoprecipitates DQ. When fractions 14/15 were immunoprecipitated for DQ, the DQβ chain was detected by western blotting (lane 2, lower panel). Strikingly, however, no DRα was coprecipitated with DQ from the gradient fractions containing MHCII-Ii complexes (lane 2, upper panel). As a control, immunoprecipitation of DR shows that DRα (upper panel) and DRβ (lower panel) are indeed present in both pools. This result suggests that in untransfected cells, complexes of DQ with Ii do not contain DR.

### Subunit composition of DR-Ii oligomers

**Figure 8:**
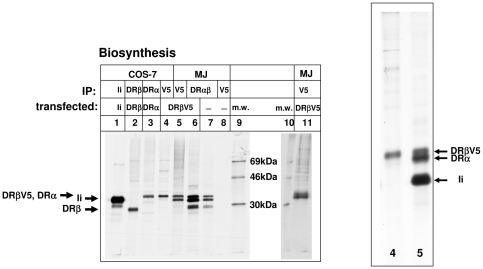
Metabolic labeling reveals that only one DRβ chain associated to an Ii homotrimer. The composition of DR-Ii complexes in pulse labeled immunoprecipitates from MelJuSo cells, stably transfected with DRβV5 encoding cDNA. COS cells and MelJuSo cells expressing MHCII subunits and Ii were pulse labeled for 10 min with ^35^S-methionine. Cells were lysed and immunoprecipitated for Ii (monoclonal antibody Bu45, lane 1), for DRβ (S35, lane 2), for DRα (monoclonal antibody TAL-1B5, lane 3), for DRβV5 (monoclonal antibody V5, lanes 4 and 5), for DRαβ (monoclonal antibody I251SB, lanes 6 and 7) and as a control of reactivity of V5 monoclonal antibody with untransfected MelJuSo cell extract (monoclonal antibody V5, lane 8). Lanes 9 and 10 show molecular weight standards. In lane 11 a DRβV5 immunoprecipitate of MelJuSo cells which were pulse labeled for 10 min with ^35^S methionine and subsequently cultured for 40 min with medium containing non-radioactive methionine is shown. Immunoprecipitates from lanes 4 and 5 (left panel) were separated on a 20 cm long SDS PAGE gel to separate DRβV5 and DRα chains (right panel).

Finally, we wished to analyze the formation of the complexes of DR and Ii, which occurs soon after biosynthesis in the ER. DRβV5-transfected MelJuSo cells were pulse labeled with ^35^S-methionine for 10 min. (At this short time point, the majority of labeled MHCII and Ii chains localize to the ER). Cells were then lysed, and DRβV5 was immunoprecipitated with anti-V5 monoclonal antibody or with the conformation-dependent monoclonal antibody I251SB against DRαβ. To assess the mobility of the individual polypeptide chains, Ii, DRβ, DRα and DRβV5 were immunoprecipitated from lysates of transfected COS-7 cells (Fig. 8, lanes 1 to 4).

Immunoprecipitation of DRβV5 from lysates of the transfected cells revealed two bands (Fig. 8, lane 5), but no band for endogenous DRβ, suggesting that as in the steady-state experiment (Fig. S2), the transfected DRβV5 was not associated with endogenous DRβ via Ii The lower band in lane 5 corresponds to the mobility of Ii, and the upper band contains both DRα and DRβV5, as shown by the separation of immunoprecipitates of lanes 4 and 5 on a long gel (right panel). Lane 5 also indicates that DRβV5 is not overexpressed in comparison to endogenous DRα.

The results of this experiment suggest that the ER form of the DRαβIi complex contains only one DRβ chain and not several. To rule out that the labeling time (ten minutes), was to short to allow for the formation of a larger complex, we pulse labeled DRβV5 expressing cells for 10 minutes with ^35^S methionine chased for 40 minutes with unlabeled methionine, and immunoprecipitated DRβV5 (lane 11). As in lane 5, two bands containing DRβV5 plus DRα and Ii were separated whereas endogenous DRβ could not be detected. Thus, the complex of DR and Ii contains only one DRβ chain.

### A revised model for MHCII-Ii oligomers

**Figure 9:**
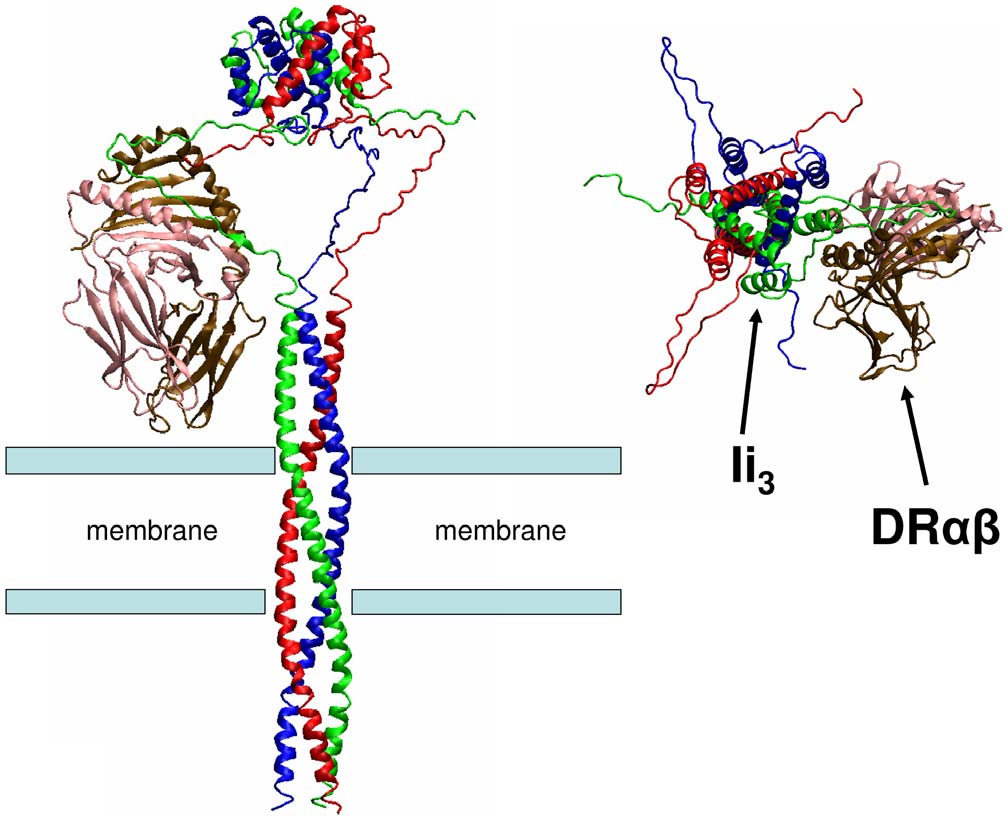
Modeling of MHCII-Ii structure. (left) Molecular model (see Methods section for details) for a pentameric MHCII-Ii oligomer consisting of a trimeric Ii (chains are in blue, red and green cartoons) and one bound MHC-II heterodimer (alpha and beta chains in pink and brown cartoon, respectively). The position of the membrane is indicated (bars representing approximately the lipid-solvent boundary). (right) Top view of the model (from the ER) along the axis of the coiled-coil structure.

Finally, we attempted to reconcile our data with the existing knowledge of the three-dimensional structure of MHCII and Ii using a computational model. At first, we considered the structure of Ii_3_. The primary sequence of Ii shows a repeat of leucine pairs, starting at the cytosolic tail at Leu-7 of the Ii 33 kDa form of the polypeptide. The leucine residues appear in a distance of seven amino acids and this motif is repeated five times through the membrane spanning region towards the luminal part up to residue 81, suggesting an extensive interhelical interaction such as a leucine zipper. Since the three Ii chains are known to interact within the membrane [32], we postulate that the trimeric domain of Ii extends from residues Leu-7 to Leu-81. Figure 9 shows a structural model in which the amino terminus of Ii shows a coiled structure composed of three Ii subunits, followed by a non-ordered sequence between amino acid 82 and the C-terminal trimeric domain. This loop contains the CLIP sequence, which fills the cleft of the MHCII heterodimer [6]. In the model, the binding of the CLIP sequence to the MHCII heterodimer is sterically possible in the complex if one assumes a slightly kinked arrangement of the MHCII molecule with respect to the membrane. The other two Ii chains are indicated in the same conformation as that associated with an MHCII heterodimer. It has been shown that the CLIP containing segment is disordered in the absence of a bound MHCII heterodimer [19]. Hence, the segment structure of the two unbound Ii-chains in the model represents one among several possible conformations. According to the structural model, binding of one MHCII molecule would not sterically exclude the possibility of two other Ii chains to adopt a near-bound conformation of the CLIP segment. However, consistent with a pentameric structure, it is possible that binding of one MHCII heterodimer restricts the conformational freedom of the Ii trimer by introducing a kink in Ii_3_, thus abolishing the binding affinity of Ii_3_ for further αβ heterodimers.

## Discussion

Shortly after biosynthesis in the ER, MHCII α and β chains form transient aggregates [33]. Expression of Ii enables the release of α and β chains from such aggregates and facilitates correct assembly of the MHCII subunits [34]. When assembly is accomplished, the MHCII-Ii complex exits the ER. The three-dimensional structure of the MHCII-Ii complex has not yet been resolved by crystallography or nuclear magnetic resonance, and thus its composition is still not fully clarified. In the initial report by Roche et al. [12], the authors concluded from crosslinking experiments that a band of lower mobility than 220 kDa molecular weight marker represented the crosslinked DR-Ii complex, and they hypothesized that the MHCII-Ii complex is composed of nine subunits, an Ii trimer and three DR heterodimers, with a total molecular weight of 260 kDa [35]. In agreement with the original observation by Roche and collaborators, we find that DR indeed crosslinks into high molecular weight complexes, but we also find that such complexes are of a very broad size range and that besides Ii (and where applicable, MHCII) they may contain other cellular components. Our results indicate that crosslinking experiments alone are not sufficient to accurately predict the size of the MHCII-Ii complex.

In this study, we have investigated the stoichiometry of MHCII-Ii complexes by using epitope-tagged subunits of MHCII and assessing their co-isolation with endogenous components. In contrast to the work cited above, we find strong evidence for a pentameric complex consisting of an Ii trimer and one dimer of MHCII.

Several lines of evidence from the literature support our findings that Ii forms distinct complexes with the MHCII isotypes. Initial studies in mice were performed with antisera specific for H-2A or H-2E isotypes of Ia (mouse MHCII) [15]. Such locus-specific antisera separately isolated metabolically labeled H-2A and H-2E isotypes bound to Ii, but did not coprecipitate H-2A with H-2E or vice versa [36]. The co-isolation of H-2A and H-2E that did occur with some monoclonal antibodies was found to come from cross-reactivity of the monoclonal antibodies to the two isotypes [37]. Likewise, in humans, the cross-reactivity of many anti-HLA-class II monoclonal antibodies with several of the three isotypes may explain why distinct complexes of DP, DQ, and DR with Ii have not been clearly demonstrated to date. We show here that DP or DQ immunoprecipitates do not contain DR subunits. This result suggests that the Ii trimer only associates with one of the DP, DQ, or DR isotype heterodimers.

The direct determination of size of the Ii-MHCII complex is naturally difficult because of the highly elongated nature of Ii, which leads to an apparent size of twice its molecular mass in gel filtration and light scattering [2], and because of the association of detergent with the protein, which might lead to an artefactual increase in the hydrodynamic radius [38]. Thus, in a combined gel filtration/sedimentation velocity ultracentrifugation experiment, a mass of 390 kDa was calculated for the DR-Ii complex [12], with the deviation of this value from the calculated mass of the nonameric Ii_3_(αβ)_3_ complex (260 kD, see above) assigned to detergent. In our experiments by separation of cell lysates in a sucrose gradient and comparison to protein standards, we estimate a molecular weight of about 130 to 160 kDa for the DR-Ii complex. Here, the sedimentation properties of the protein are not entirely determined by its association with detergent micelles, since we can very well separate small DRαβ from large DRαβIi complexes (Figures 1, 2 and 3).

To investigate whether the use of a different detergent, Triton X-100, was responsible for our differing size estimate, we examined two additional detergents, n-octylglycoside and digitonin for separation of MHCII complexes in a sucrose gradient. In our hands, the sucrose gradient with either of these detergents did not separate peptide loaded MHCII molecules from MHCII-Ii complexes (data not shown). To show that the use of Triton X-100 did not lead to dissociation of a larger complex, we then demonstrated that Ii-DR complexes from digitonin-lysed cells are indeed stable in Triton X-100 (Fig. S1F). We therefore conclude that Triton X-100, which can be used to separate MHCII-Ii complexes from Ii free MHCII heterodimers, allows the isolation of intact and complete Ii-DR complexes.

Nuclear magnetic resonance studies of recombinant soluble Ii have shown that the MHCII binding site is contained in a disordered region of Ii [39] that acquires an ordered conformation upon MHCII binding [19]. *In vitro* assembly of titrated soluble Ii with constant amounts of soluble DR (truncated just before the transmembrane domain) showed additional bands in a non-denaturing gel at decreasing Ii concentrations. This result was taken to suggest that Ii oligomers associate with several pairs of MHCII subunits [19]. Our data, in contrast indicate that only one full-length MHCII heterodimer is contained in the MHCII-Ii oligomer, resulting in a pentameric Ii_3_αβ complex.

Our molecular model of the complex (Fig. 9) is not per se evidence for a pentameric complex, but it conclusively demonstrates that the pentameric model is compatible with all known structural data, and it suggests a possible mechanism for unique binding of a MHCII heterodimer to Ii_3_. We postulate that upon binding of a single MHCII heterodimer to one unstructured CLIP sequence, the triple-helical core domain of Ii_3_ changes its conformation, which imposes a steric constraint onto the entire complex, discouraging binding of further MHCII heterodimers.

Interestingly, the murine MHC class I molecule H-K^b^, which is structurally related to MHCII molecules, was suggested to adopt a supine (lying down) orientation on the membrane, with the peptide binding site close to the membrane (while remaining accessible to the T cell receptor and the CD8 coreceptor [40]). If an MHCII αβ heterodimer adopted a similar conformation, then Ii would need to bent over extremely strong to interact with them, which would presumably prevent the binding of a second and third MHCII αβ heterodimer. Conclusive evidence to the nature and degree of the conformational change of Ii_3_ upon MHCII αβ binding will probably only be obtained upon the resolution of the three-dimensional structure of the complex.

Why would Ii itself form a trimer if it does not bind three MHCII heterodimers? We can offer several hypothetical explanations. First, Ii contains several topogenic sequences in its cytosolic tail that are responsible for internalization from the cell surface and for the targeting of the complex to endosomes [41]. Trimerization of Ii is required for the efficient targeting of MHCII-Ii to endosomal compartments [42]–[43], suggesting that the trimeric character allows Ii to fulfil its function in targeting MHCII to endosomes more efficiently. The trimeric tail could have a similar function in ER-to-Golgi transport, where COPII coat of transport vesicles is known to recognize oligomeric cytosolic tails of proteins, and/or in other intracellular steps [44]–[45]. Second, Ii (CD74) plays a role as a cell surface receptor or coreceptor for a number of proteins, most prominently for the macrophage inhibitory factor (MIF). Since MIF itself is a trimer with rotational symmetry, a trimerization of Ii might be required to bind MIF [27], [46].

A pentameric MHCII- Ii complex has some distinct advantages for the process of MHCII antigen presentation. First, one would assume that due to the unordered nature of the MHCII- binding domains of Ii, a pentamer would be more sensitive to proteolytic digestion in the endosome; two of the three Ii molecules would be rapidly cleaved, leading to the breakup of the Ii_3_ trimer. In a nonamer, the protease-resistent MHCII heterodimers would shield Ii from degradation in endocytic compartments. Second, in a pentameric complex, ER quality control of MHCII would probably be faster and more efficient. Usually, two alleles each of the α and β chains of DP and DQ isotypes are expressed (DRα is nonpolymorphic). From the numerous combinations of DP, DQ and DR α and β chains that are present in the ER, only a few can form matching heterodimers. The quality control in the ER permits exit of MHCII-Ii oligomers only after assembly of the matching MHCII chains, while mismatched αβ heterodimers do not exit the ER [47]. The assembly of these matched heterodimers takes place on Ii_3_ [48]. If a nonameric complex was formed in the ER, three matched dimers would have to be found for the complex to leave the ER [49], whereas association of the Ii trimer with only one αβ heterodimer would allow the immediate exit of a MHCII molecule from the ER, as soon as it is properly assembled. Substantial work is required to explore the cell biological consequences of our findings.

## Materials and Methods

### Cells, transfection, and biosynthetic labelling

MelJuSo is a human melanoma cell line that expresses large amounts of HLA class II molecules and Ii. Monocyte-derived DCs were a kind gift of Dr. S. Koch, Determatology Department, Bonn. MelJuSo cells, Raji cells and COS-7 cells were cultured in DMEM containing 10% FCS. Cells were transfected with JetPEI (QBiogen, Heidelberg, Germany) according to instructions of the supplier. Briefly, 1 to 2 µg DNA were mixed with 2.5 to 5 µl JetPEI in 100 µl medium, added to 5×10^5^ cells, and subsequently the cells were cultured for three days. For labeling, 2×10^6^ cells were suspended in 200 µl RPMI medium without methionine and 100 µCi of ^35^S methionine were added. After a pulse of 10 minutes, the cells were pelleted by centrifugation and washed with cold PBS. The pulse labeled cells were chased with medium containing non-radioactive methionine.

### Immunoprecipitation, western blotting, and immunofluorescence microscopy

Cells were lysed with 1% NP40 or with 1% Triton X-100 in Tris-buffered saline pH 7.4 containing protease inhibitors (Complete, Roche Diagnostics, Mannheim Germany). DNA and debris were removed by high speed centrifugation. Pre-absorption of the lysate was conducted with CL4B-Sepharose (Amersham, Freiburg, Germany). Immunoprecipitates were isolated with protein A Sepharose, separated by SDS-PAGE, and blotted onto Immobilon P membrane (Millipore, Schwalbach, Germany). Immunoblotting was conducted with primary and secondary antibodies conjugated with horseradish peroxidase and subsequently visualized with ECL enhanced chemoluminescence. Western blots were calibrated with protein standards. To assess sensitivity of the assay, titration experiments were conducted. We determined that cell lysates of 30 000 cells were sufficient to detect MHCII subunits or Ii. In our experiment, we used lysates of 1 million cells for IP and 100 000 cells as lysate controls. The exposure time of the western blots was usually 1 to 5 min. For endoglycosidase treatment, cell lysates were digested with EndoH (200 U) and with PNGase F (100 U) overnight at 37°C as recommended by the supplier (NEB Biolabs, Frankfurt, Germany). For confocal immunofluorescence microscopy, cells were seeded on coverslips, fixed with 4% PFA, and permeabilized with 1% Triton X-100. Cells were labeled with CD63 Alexa 488 and with V5 monoclonal antibodies followed by Alexa-594 secondary antibody. Staining was monitored by confocal-fluorescence microscopy (LSM-510 Meta, Zeiss). The size of the optical section was 1 µm.

### Molecular Modeling

A molecular model of a pentameric MHCII-Ii oligomer consisting of a trimeric MHCII-Ii and one bound MHCII heterodimer was generated by combining known experimental structures of a CLIP peptide in complex with HLA-DR3 (pdb1A6A) [6], the known C-terminal trimerisation domain of Ii (residues 118 to 216, pdb1IIE, [3]) and a homology model for the N-terminal coiled-coil structure (residues 7-81) using the SPDBViewer [50] and the Amber molecular modelling software [51]. The N-terminal trimeric coiled-coil structure was built using a known structure (pdb3MO6) as template guided by the leucine repeats in the Ii N-terminal sequence. The connecting loop segments between Ii segments were generated with the SPDBViewer [50]. The subunits were placed manually avoiding steric overlap and allowing insertion of missing loop segments between the C-terminal trimerisation domain, the CLIP sequence and the N-terminal coiled-coil structure employing the loop builder option in SPDBViewer. It is important to emphasize that the structural model represents only one sterically possible structure among many possible alternative loop conformations and variations in the relative subunit arrangement. The complete model was finally energy minimized to remove any sterical overlap employing the Amber package [51].

### DNA constructs and antibodies

DPα (DPA1*0103), DPβ (DPB1*0401) and DQA1*0103 were cloned from dendritic cells generated from blood monocytes. The DP α and β chains were appended with Ab-tags [52]. The DRβ (DRB1 0101) chain was tagged with V5-His epitopes (pcDNA3.1 Invitrogen, Karlsruhe, Germany). DRα and DQβ contain appended V5 epitopes. TAL-1B5 monoclonal antibody [53] is directed against the cytoplasmic tail of DRα. It was used for immunoprecipitation and for western blotting. Monoclonal antibody I251SB [54] is directed against DRαβ heterodimers. It does not react with single DR chains. Monoclonal antibodies ISCR3 and 16.23 against DR have been described previously [55]–[56]. Serum 35 (S35) was generated by immunizing a rabbit with a DRβ fusion protein [57]. Monoclonal antibody LGII-612.14 reacts to DR, DP and DQβ chains (purchased from Dr. S. Ferrone, NY). Monoclonal antibody1a3 is specific for DQ and was purchased from MyBioSource (San Diego, CA). Monoclonal antibody Bu43 is directed against the C-terminus, Vic-Y1 to the N-terminus and Bu45 to the trimerisation domain of Ii [58]. V5 and His monoclonal antibodies were purchased from Invitrogen.

### Sucrose gradient ultracentrifugation

The sucrose gradient was poured by gradually mixing 0.4 M with 0.8 M sucrose in 10 mM Tris-buffered saline pH 7.5 containing 0.5% Triton X-100. One ml of Raji cell lysate was placed on top of the gradient and centrifuged at 100.000 g for 50 h. Twenty-four fractions of the gradient were subsequently collected from the top of the tube. The sucrose concentration of the fractions was determined with a refractometer (Fig. 1A. upper panel). By separation of protein standards and subsequent analysis of the fractions by SDS PAGE and Coomassie blue staining, a size calibration of the gradient was achieved (Fig. 1A, lower panel). The protein standard IgG (m.w. 150 kDa), which defines a region with higher molecular weight, was identified in fractions 13 to 16 (see insert Fig. 1A, lower panel). In Figure 1B, upper panel, samples obtained from separation of Raji cell lysates were mixed with reducing SDS sample buffer, boiled, separated by SDS PAGE and immunoblotted for DRα. The gradient was reproduced in more than ten experiments, showing no more variation than one fraction.

### Chemical crosslinking

To 100 µl of a pool of fractions 7 and 8 or of fractions 14 and 15 of the sucrose gradient (without Tris) separated Raji cell lysates increasing amounts of the thiol- cleavable crosslinker 3,3′-dithiobis [succinimidyl propionate] (DSP) were added. Samples were incubated for 2 h on ice. For quenching the excess crosslinker, 10  µl of 1 M Tris was added. Samples were incubated with non-reducing sample buffer, boiled for 5 min, and subjected to SDS-PAGE.

## Supporting Information

Figure S1 **Carbohydrate processing, peptide binding of transgenic MHCII molecules and stability of MHCII-Ii complexes.** A) MelJuSo cells were stably transfected with a V5-tagged DPβ encoding cDNA. The lysate of the DPβ–transfected MelJuSo cell clone was treated with PNGase F (lane 2), or with EndoH (lane 3). The digested (lane 2 and 3) and non-digested (lane 1) lysates were immunoblotted for DPβV5 chain. B) Intracellular transport of DRβV5 was monitored by digestion of cell lysate with PNGase F (lane2), or with EndoH (lane 3). Non-digested cell lysate was separated in lane 1. Lysates from non-transfected cells were analyzed in lanes 4 to 6. SDS-PAGE separated lysates were blotted for DRβV5 (lanes 1 to 3) and for DRβ (lanes 4 to 6). C) Cell lysate from DRβV5-transfected MelJuSo cells was incubated for 1 h in SDS sample buffer at RT (lane 1). Lane 2 shows cell lysate boiled for 5 min in SDS buffer. The SDS separated lysates were immunoblotted for DRβV5. The position of the peptide-bound DRαβV5 heterodimer and of the dissociated DRβV5 chain is indicated. D) MelJuSo (MJ) and MJDRβV5 cells were stained with CD63-Alexa 488 and with V5 monoclonal antibodies and examined by confocal-immunofluorescence microscopy. The left panels show CD63 and the middle panels V5 staining. In the right panel the two patterns were merged. E) MJDRαV5 cells were lysed and immunoprecipitated with V5 mAb. The immunoprecipitate (lane 1) and the lysate (lane 2) were separated by SDS PAGE and western blotted with mAb Bu43 for Ii (notice that cell lysate contains DR-associated Ii and an excess of free Ii). The position of Ii and of H and L chains of mAb V5 are shown on the left. F) Raji cells were lysed in 1% digitonin and Ii was immunoprecipitated with mAbs Vic-Y1 and Bu45. Immunoprecipitates were washed with digitonin buffer and incubated with 1% Triton X-100. The immunoprecipitate (lanes 1 and 3) and the Triton X-100 supernatant (lanes 2 and 4) were western blotted for Ii (lanes 1 and 2) (mAb Bu43) and for DRα (lanes 3 and 4) (mAb TAL-1B5). The positions of Ii, of DRα and of immunoglobulin H and L chains are indicated by arrows.
(TIF)

Figure S2 **Isolation of DRβV5 from transfected MelJuSo cell lysates.** A) Lysates from the DRβV5-transfected MelJuSo cell line were immunoprecipitated for DRβV5 (monoclonal antibody V5, lane 1), for DRβHis (monoclonal antibody His, lane 2), for the DRαβ heterodimer (monoclonal antibody I251SB, lane 3), and for DRα (monoclonal antibody TAL-1B5, lane 4). Lysates from DRβV5 and from untransfected MelJuSo cells were separated in lanes 5 and 6. Three parallel blots were immunostained for DRβ (S35, upper panel), for DRβV5 (monoclonal antibodyV5, middle panel) and for DRα (monoclonal antibody TAL-1B5, lower panel). Arrows indicate the positions of DRβ, of DRβV5 and of DRα. B) DRβV5 and untransfected MelJuSo cells (MJ) were lysed in 1% digitonin. DRαβ was immunoprecipitated by the conformation-dependent mAb ISCR3. Immunoprecipitates and cell lysates were SDS-PAGE separated and western blotted for DRβ (left panel, mAb LGII-612.14), for DRα (middle panel, mAb TAL-1B5), or for Ii (right panel, mAb Bu43). The positions of DRβV5, DRβ, DRα and of Ii are indicated by arrows.
(TIF)

Text S1 Supplemental text.
(DOC)
