# Supplementary figures and images for: Stoichiometry of HLA Class II-Invariant Chain Oligomers
Source: PLoS One. 2011 Feb 22;6(2):e17257. doi: 10.1371/journal.pone.0017257 (PMC3043101; doi:10.1371/journal.pone.0017257)

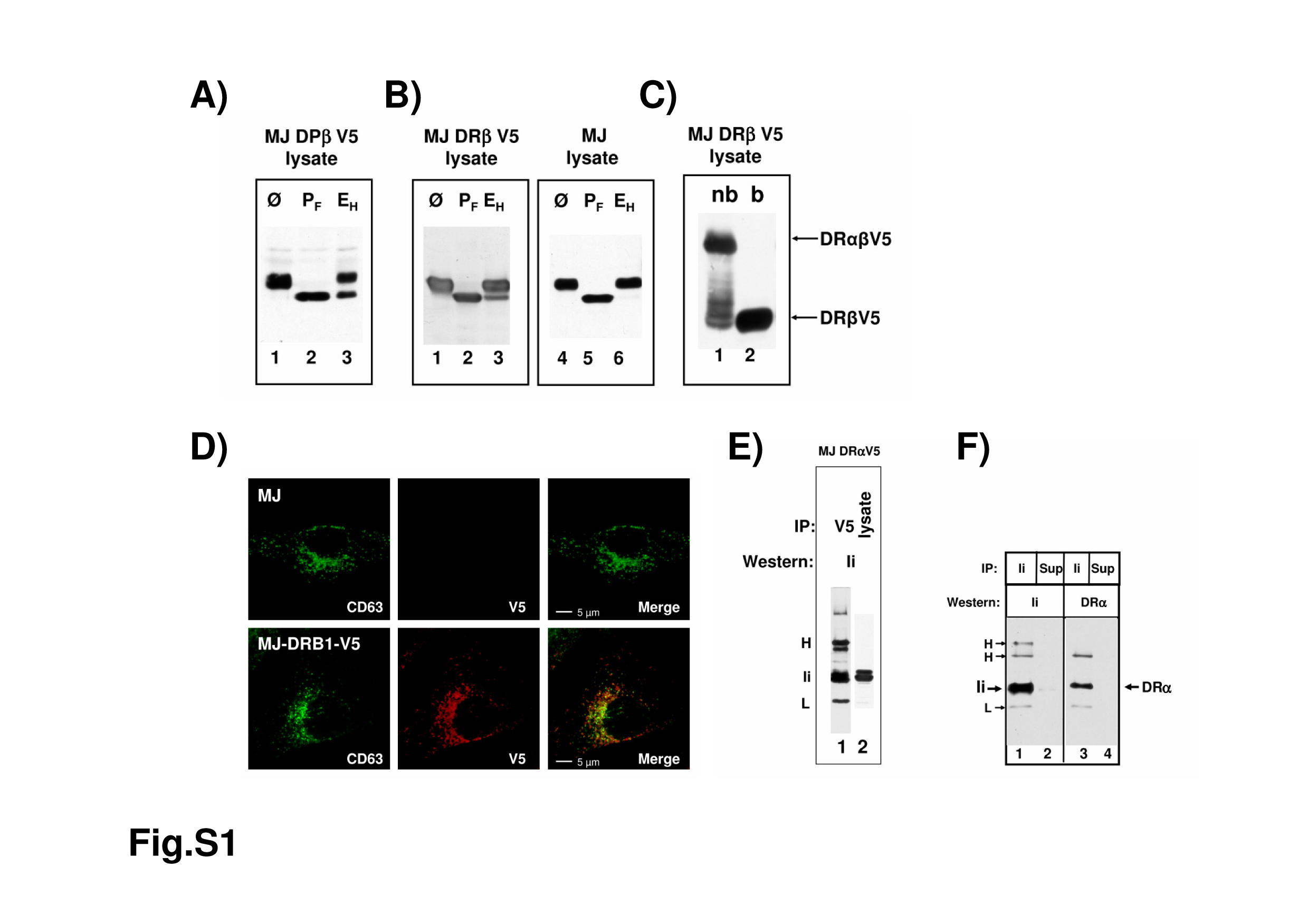

Supplement: Figure S1 — Carbohydrate processing, peptide binding of transgenic MHCII molecules and stability of MHCII-Ii complexes. A) MelJuSo cells were stably transfected with a V5-tagged DPβ encoding cDNA. The lysate of the DPβ–transfected MelJuSo cell clone was treated with PNGase F (lane 2), or with EndoH (lane 3). The digested (lane 2 and 3) and non-digested (lane 1) lysates were immunoblotted for DPβV5 chain. B) Intracellular transport of DRβV5 was monitored by digestion of cell lysate with PNGase F (lane2), or with EndoH (lane 3). Non-digested cell lysate was separated in lane 1. Lysates from non-transfected cells were analyzed in lanes 4 to 6. SDS-PAGE separated lysates were blotted for DRβV5 (lanes 1 to 3) and for DRβ (lanes 4 to 6). C) Cell lysate from DRβV5-transfected MelJuSo cells was incubated for 1 h in SDS sample buffer at RT (lane 1). Lane 2 shows cell lysate boiled for 5 min in SDS buffer. The SDS separated lysates were immunoblotted for DRβV5. The position of the peptide-bound DRαβV5 heterodimer and of the dissociated DRβV5 chain is indicated. D) MelJuSo (MJ) and MJDRβV5 cells were stained with CD63-Alexa 488 and with V5 monoclonal antibodies and examined by confocal-immunofluorescence microscopy. The left panels show CD63 and the middle panels V5 staining. In the right panel the two patterns were merged. E) MJDRαV5 cells were lysed and immunoprecipitated with V5 mAb. The immunoprecipitate (lane 1) and the lysate (lane 2) were separated by SDS PAGE and western blotted with mAb Bu43 for Ii (notice that cell lysate contains DR-associated Ii and an excess of free Ii). The position of Ii and of H and L chains of mAb V5 are shown on the left. F) Raji cells were lysed in 1% digitonin and Ii was immunoprecipitated with mAbs Vic-Y1 and Bu45. Immunoprecipitates were washed with digitonin buffer and incubated with 1% Triton X-100. The immunoprecipitate (lanes 1 and 3) and the Triton X-100 supernatant (lanes 2 and 4) were western blotted for Ii (lanes 1 and 2) (mAb Bu43) and for D [file pone.0017257.s001.tif]

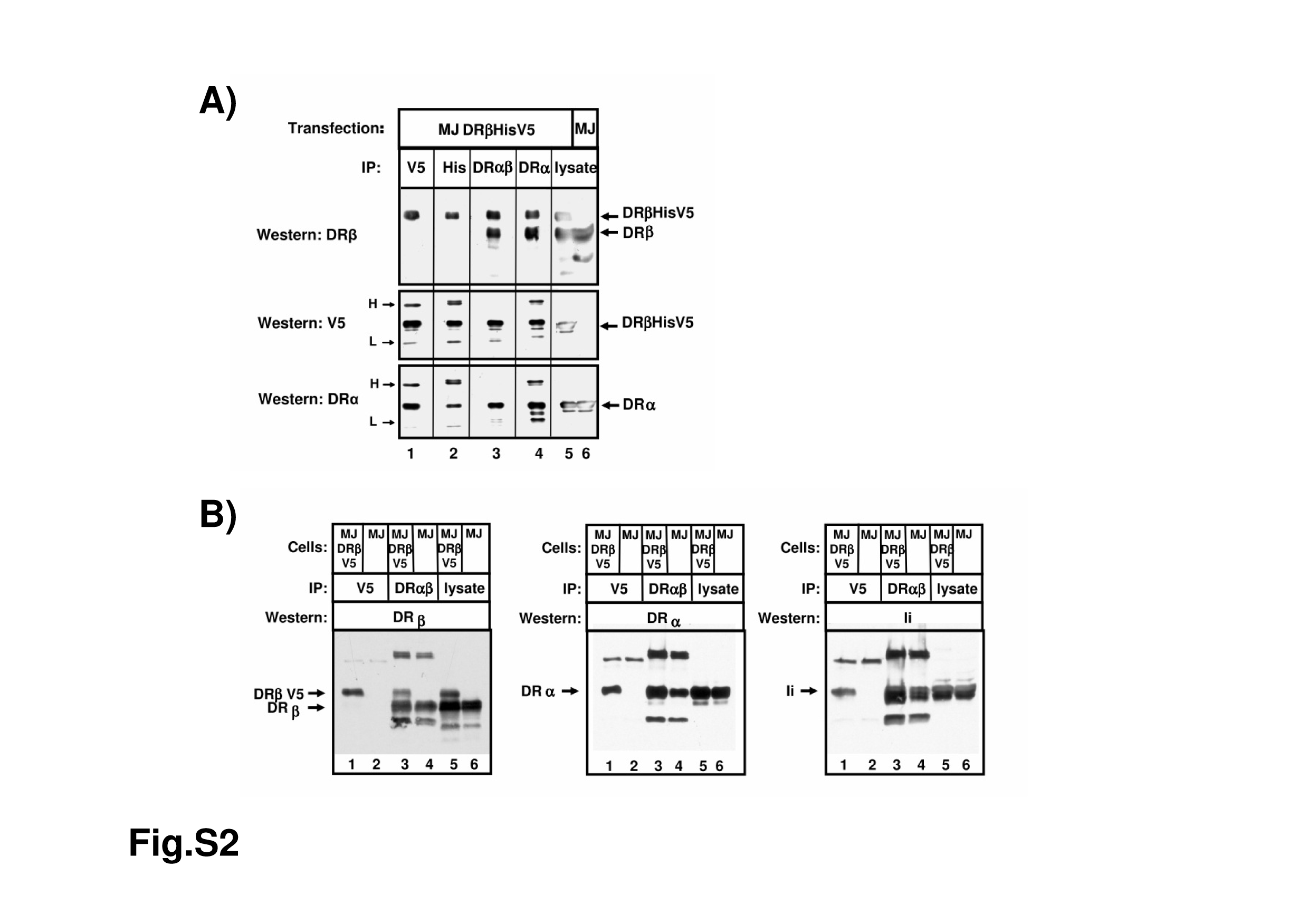

Supplement: Figure S2 — Isolation of DRβV5 from transfected MelJuSo cell lysates. A) Lysates from the DRβV5-transfected MelJuSo cell line were immunoprecipitated for DRβV5 (monoclonal antibody V5, lane 1), for DRβHis (monoclonal antibody His, lane 2), for the DRαβ heterodimer (monoclonal antibody I251SB, lane 3), and for DRα (monoclonal antibody TAL-1B5, lane 4). Lysates from DRβV5 and from untransfected MelJuSo cells were separated in lanes 5 and 6. Three parallel blots were immunostained for DRβ (S35, upper panel), for DRβV5 (monoclonal antibodyV5, middle panel) and for DRα (monoclonal antibody TAL-1B5, lower panel). Arrows indicate the positions of DRβ, of DRβV5 and of DRα. B) DRβV5 and untransfected MelJuSo cells (MJ) were lysed in 1% digitonin. DRαβ was immunoprecipitated by the conformation-dependent mAb ISCR3. Immunoprecipitates and cell lysates were SDS-PAGE separated and western blotted for DRβ (left panel, mAb LGII-612.14), for DRα (middle panel, mAb TAL-1B5), or for Ii (right panel, mAb Bu43). The positions of DRβV5, DRβ, DRα and of Ii are indicated by arrows. (TIF) [file pone.0017257.s002.tif]
