# Supplementary material for: Stoichiometry of HLA Class II-Invariant Chain Oligomers
Source: PLoS One. 2011 Feb 22;6(2):e17257. doi: 10.1371/journal.pone.0017257 (PMC3043101; doi:10.1371/journal.pone.0017257)
Supplement: Text S1 — Supplemental text. (DOC) [file pone.0017257.s003.doc]

**Text S1,**

**SUPPORTING INFORMATIONS**

**Assembly and transport of transgenic MHCII subunits indicates association with endogenous subunits**

We expressed V5-epitope tagged DQα, DPβ, DRα, or DRβ in MelJuSo cells to examine the composition of the formed complexes (Fig. S1) and the transport of the transgenic MHCII subunits in transfected cells.

To achieve a constant level of expression of the transfected MHCII cDNA, we produced a MelJuSo cell line by stable transfection of DPβV5. At first, we inspected the fate of DPβV5 in the transfected cells. We examined the intracellular transport of the transgenic DPβV5 chain by monitoring carbohydrate maturation. EndoH (Endoglycosidase H) digestion distinguishes complex type (EndoH-resistant) from high mannose type carbohydrates (EndoH-sensitive). Cell lysates of transfected MelJuSo cells were left untreated (Fig. S1A, lane 1), digested with EndoH (lane 3), or with PNGase F (lane 2), which completely removes the sole N-linked carbohydrate on DPβ chain. The complex type carbohydrate, which is acquired during transport of DP through Golgi compartments, remains uncleaved by EndoH treatment.

Figure S1 A shows that a substantial proportion of the transgenic DPβV5 chain carries EndoH resistant carbohydrates (lane 3), that co-migrates in the position of untreated DPβV5 chain (lane 1). Single expressed MHCII chains are retained in the ER. Therefore, the monitored carbohydrate maturation indicates assembly of the transgenic β chain with endogenous α chain. Next, we examined the localization of a transgenic MHCII subunit in endocytic vesicles by co-localization with the tetraspanin CD63. Figure S1D shows staining of MelJuSo and of MelJuSoDRβV5 cells with CD63 and with V5 monoclonal antibodies. The superimposed label (right patterns) indicates that the endosomal marker CD63 and DRβV5 are co-stained, indicating colocalization in endosomes.

In a second approach, a DRβ chain tagged with a V5-His epitope was stably expressed in MelJuSo cells. At first, carbohydrate maturation of DRβ in transfected MelJuSo cell lysates was inspected. Figure S1B (left panel) (lanes 1 to 3) shows an EndoH resistant DRβV5 band accompanied by a small amount of EndoH sensitive DRβ. The pattern of endogenous digested DRβ is shown in lanes 4 to 6. In addition, we examined whether the transgenic DRβV5 chain formed SDS-resistant complexes of DRα and β chains with bound peptide. The peptides and the DR subunits dissociate after boiling in SDS sample buffer. The right panel of Fig. S1C shows non-boiled and boiled cell lysates, which were immunoblotted for DRβV5. Upon boiling, the slowly migrating DRβV5 band in lane 1, consisting of pDRαDRβV5 is converted to a fast migrating band corresponding to the position of single DRβV5 chain (lane 2). In addition, we demonstrate co-isolation of Ii with DRαV5, which is required to examine co-isolation of endogenous DRα. An immunoprecipitate of the V5-tagged DRα chain from lysates of MelJuSoDRαV5 cells and subsequent western blotting for Ii is shown in Fig. S1E. Equivalent results were obtained by immunoprecipitation of DRβV5 or DPβV5 from stably transfected MelJuSo lines and immunoblotting for Ii (data not shown).

Fig. S1F demonstates the stability of DR-Ii complexes in the detergent Triton X-100 in comparison to digitonin. Raji cells were lysed in 1 % digitonin and immunoprecipitated with mAbs Vic-Y1 and Bu45. Vic-Y1 is directed to the N terminus and Bu45 detects the trimerisation domain of Ii. The two mAbs were employed to increase the avidity of the Ag-Ab complexes to Protein G Sepharose. Protein G Sepharose was used to achieve binding of the IgG1 mAb Bu45. The immunoprecipitates were washed with buffer containing digitonin and subsequently incubated with 1 % Triton X-100. Lanes 1 and 3 show the immunoprecipitates blotted for Ii or for DRα, indicating immunoisolation of Ii and of co-isolated DRα. In lanes 2 and 4 the Triton X-100 supernatant was blotted for Ii and for DRα. Lane 2 shows traces of Ii and of immunoglobulin H chain bands. These bands were produced by release of small amounts of Ii-Ab complexes from the Protein G Sepharose. The Triton X-100 supernatant blotted for DRα did not show a specific band. If DR heterodimers bound to the Ii trimer were sensitive to the detergent Triton X-100, the release of the α-β heterodimer would have been detected as a strong DRα band in lane 4.

**DRβ does not co-isolate with DRβV5**

To confirm that only one DR heterodimer is contained in a DR-Ii complex, the content of β chains in the oligomer was examined. If the transgenic DRβV5 chain expressed in MelJuSo cells is contained in a complex composed of an Ii trimer and two or three DR heterodimers, it should be possible to co-isolate the endogenous DRβ chains via the DRβV5 chain. The transfected DRβV5 chain, which in addition to V5 contains a His epitope, was immunoprecipitated with anti V5 or with anti His monoclonal antibodies. (Fig. S2A, lanes 1 and 2). In lanes 3 and 4, DR was immunoprecipitated with the conformation-dependent DR monoclonal antibody I251SB, which reacts to αβ heterodimers, or with TAL-1B5 monoclonal antibody against DRα. Immunoblotting of lysates from DRβV5 transfected MelJuSo and from non-transfected MelJuSo cells demonstrates transgenic and endogenous DRβ. Three parallel blots were immunoblotted for transgenic and for endogenous DRβ (upper panel), for DRβV5 (middle panel), and for DRα (lower panel).

The endogenous DRβ chain was not co-isolated with the transfected DRβ polypeptide (upper panel, lanes 1 and 2). In the lower panel, the V5- and the His-immunoprecipitated β chains stained for co-isolated DRα, indicating that DRβV5 was associated to endogenous DRα. Immunoprecipitation of DRαβ heterodimers (lane 3) and of DRα (lane 4) showed isolation of the transgenic and of the endogenous DRβ chains (upper panel). Staining of these immunoprecipitates for V5 (middle panel) or for DRα (lower panel) exhibits isolation of DRβV5 and of DRα. The presence of the transfected DRβV5, and the position of endogenous DRβ and DRα chain bands was inspected by immunoblotting of cell lysates in lanes 5 and 6.

To confirm that endogenous DRβ cannot be co-isolated with DRβV5, we repeated the experiment by using the mild detergent digitonin (Fig. S2B). MelJuSo cells transfected with DRβV5 and untransfected cells were lysed in digitonin and DR was immunoprecipitated from cell lysates by using mAbs specific for V5 or for DRαβ dimers. Western blots of the immunoprecipitates and of cell lysates are shown in Fig. S2B. The left panel of Fig. S2B demonstrates that the V5 mAb isolates DRβV5 (lane 1), but no endogenous DRβ. Lanes 3 to 6 show isolation and expression of DRβV5 and of endogenous DRβ. The middle and right panel of Fig. S2B exhibits controls blotted for DRα and for Ii. Since the endogenous DRβ chain can only be isolated by DRαβ or by DRα specific monoclonal antibodies and not co-isolated with V5-tagged DRβ, the data in Fig. S2 suggest that the endogenous and the transfected DRβ chains are not contained in one complex.
